# Supplementary material for: Overexpression of REST Represses the Epithelial–Mesenchymal Transition Process and Decreases the Aggressiveness of Prostate Cancer Cells
Source: Int J Mol Sci. 2024 Mar 15;25(6):3332. doi: 10.3390/ijms25063332 (PMC10970577; doi:10.3390/ijms25063332)
Supplement: Supplementary file 1 [file ijms-25-03332-s001.zip › ijms-2879067-supplementary.pdf]

Supplementary Material:

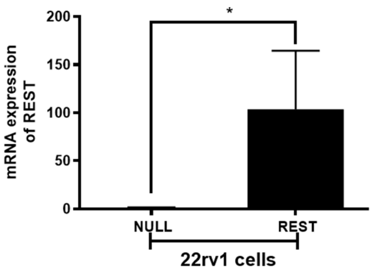

(a)

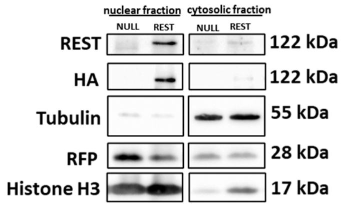

(b)

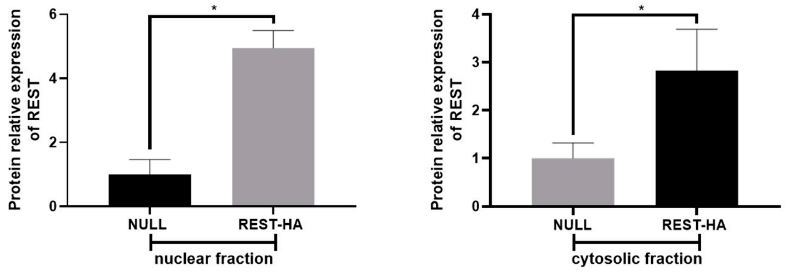

(c)

Supplementary Figure S1. **Overexpression of REST in the 22rv1 cell line.** (**a**) Levels of REST transcripts in the 22rv1 cell line transduced with overexpression vector, and in the control line (NULL); (**b**) Protein expression of REST, HA and RFP in nuclear and cytosolic fractions of the 22rv1 REST-HA and NULL cell line; (**c**) Graphical representation of REST protein levels in nuclear (left) and cytosolic (right) fractions of 22rv1 REST-HA and NULL cells. Mann-Whitney U test, p values <0.05 are considered significant, N=4.

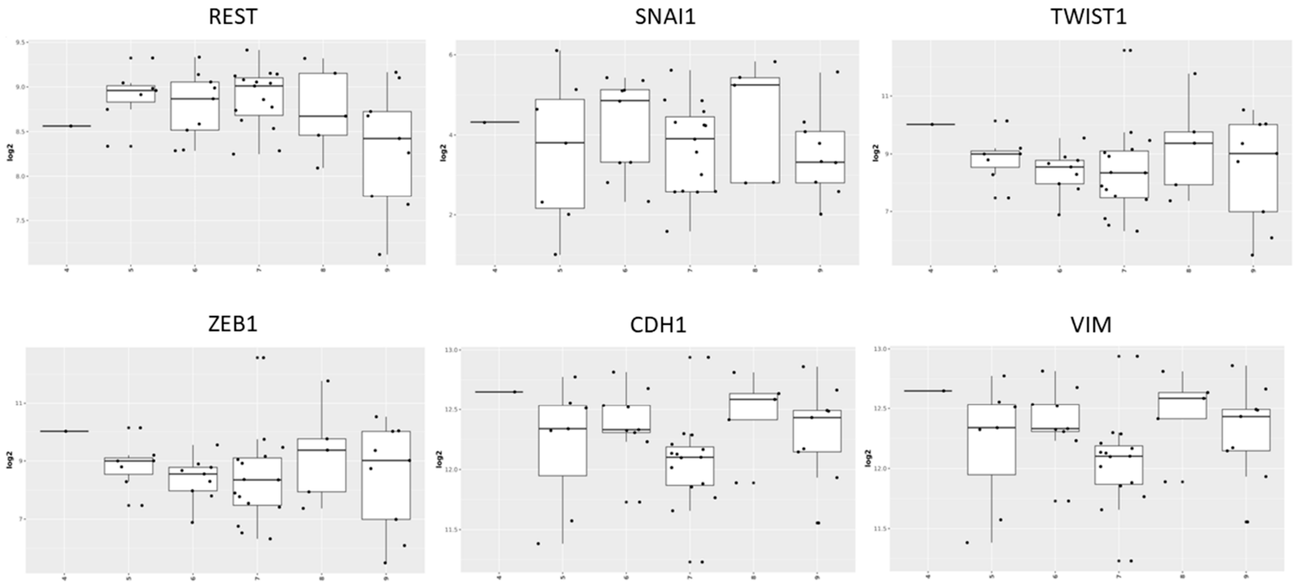

Supplementary Figure S2: Boxplot for REST, SNAI1 (Snail), TWIST1, ZEB1, CDH1 (E-cadherin) and VIM (vimentin) expression profile across prostate cancer subtypes. X axis represents Gleason Scores. Data was obtained from Gene Expression database of Normal and Tumor tissues (GENT2, http://gent2.apex.kr/gent2/).

Supplementary Table S1. Sequences of primers used for gene expression determination.

Gene name	Forward primer (5′– to 3′)	Reverse primer (5′– to 3′)
AR	CAGACCCTGAAGAAAGGCTGA	TGGAGGGAGTCAGCAATCAAG
ARv7	GTCCATCTTGTCGTCTTCGGA	GTCAGCCTTTCTTCAGGGTCT
CDH1	GAACGCATTGCCACATACAC	ATTCGGGCTTGTTGTCATTC
GAPDH	AGGGGCCATCCACAGTCTTC	AGAAGGCTGGGGCTCATTTG
KLK3	GTCCTCACAGCTGCCCAG	AGCTGTGGCTGCCTGAAAT
REST	TGCGTACTCATTCAGGTGAGA	TCTTGCATGGCGGGTTACTT
SNAI1	TTCCAGCAGCCCTACGACCAG	GCCTTTCCCACTGTCCTCATC
SNAI2	CTCCATTCCACGCCCAGCTAC	AGCCACTGTGGTCCTTGGAG
TWIST1	GGAGTCCGCAGTCTTACGAG	TCTGGAGGACCTGGTAGAGG
VIM	GCCAAGGCAAGTCGCG	GCCAAGGCAAGTCGCG
ZEB1	TTCACAGTGGAGAGAAGCCA	GCCTGGTGATGCTGAAAGAG
